# Effects of Varied Housing Density on a Hybrid Mouse Strain Followed for 20 Months

**DOI:** 10.1371/journal.pone.0149647

**Published:** 2016-02-22

**Authors:** Beverly Paigen, Joanne M. Currer, Karen L. Svenson

**Affiliations:** The Jackson Laboratory, 600 Main Street, Bar Harbor, Maine, 04609, United States of America; IGBMC/ICS, FRANCE

## Abstract

To evaluate the effect of increased housing density in a hybrid mouse strain, we evaluated a panel of physiological and behavioral traits in animals that were housed in groups of 3, 5, 8, or 12, using cages that provide 78.1 in^2^ of floor space. Such groupings resulted in cage densities that ranged from half to almost twice the density recommended by the *Guide for the Care and Use of Laboratory Animals*. While previous studies have investigated physiological effects of increased housing density using inbred mouse strains, including C57BL/6J and 129S1/SvImJ, this study tested an F1 hybrid population of C57BL/6J x 129S1/SvImJ for changes resulting from either decreased or increased housing density. Mice were followed until they were 20 months old, a substantially longer duration than has been used in previous density studies. We evaluated mortality, growth, home cage behavior, blood pressure, body composition, clinical plasma chemistries, immune function, and organ weights (heart, kidney, adrenal glands, and testes) as endpoints of chronic stress that may arise from sub-optimal housing conditions. Few statistically different parameters were observed in this study, none of which describe chronic stress and all within normal physiological ranges for research mice, suggesting that this hybrid strain was not adversely affected by housing at twice the density currently recommended.

## Introduction

The 1996 *Guide for the Care and Use of Laboratory Animals* (*Guide*) [1] specified the amount of floor space that should be allocated to each mouse in a cage: an adult mouse of 15–25 gm required at least 12 in^2^ (77.4 cm^2^), and a mouse weighing over 25 gm required 15 in^2^ (98.6 cm^2^). These standards were based on the best professional judgment because experimental data about optimal space requirements were lacking. The *Guide* recognized the lack of adequate empirical data to support these guidelines and encouraged further research on data-driven alternatives to these recommendations.

In the last few decades, several improvements in animal husbandry practices have resulted in cleaner caging and animal rooms and have even lead to the establishment of many specific pathogen free facilities. Widespread use of individually ventilated cages provides animals with drier cages and better air quality. These steps have also improved conditions for animal handlers, resulting in reduced incidence of animal-related allergies in research and production settings [2]. These improvements suggested a re-evaluation of housing density recommendations. After the 1996 *Guide* was published, a number of studies evaluated mice at densities greater than those recommended by the *Guide*. Studies published through 2011 have been reviewed [3–6], and several additional studies have been published [7–15]. Several of these studies indicated that a higher density of mice per cage was not harmful and suggested that higher housing densities may even be beneficial to or be preferred by mice. Mice showed less aggression when floor space per mouse was reduced, particularly for male mice [14, 15]. Reduced aggression led to better survival [10, 11] and fewer bite wounds [7]. Mice housed more densely were less stressed, as assessed by mortality, behavior, immune function, adrenal weight, kidney weight, and heart rate [9–18]. Overall, the studies that examined the effects of housing animals at different densities concur that the well-being of mice is not compromised when they are housed at densities that are higher than were suggested by the *Guide*. However, the 2012 edition of the *Guide* [19] did not change its recommendations except to state that more animals per cage could be used if choices were based on sound data. The studies on increased density of mice published since the 1996 *Guide* evaluated many inbred strains, but no studies used hybrid strains. Because hybrid mice are often somewhat larger and more vigorous than inbred strains, we asked whether such mice would be more sensitive to increased housing density.

Previously, we tested the effects of increased housing density in B6 and found that heart rate and adrenal weight were significantly reduced in mice housed at twice the density recommended by the *Guide* [12]. We then surveyed 5 inbred strains, including B6 and 129, housed at up to 3 times the recommended density. We measured 27 parameters and found that, with increasing housing density, kidney weight decreased to some degree for all strains and adrenal weights were significantly reduced in 129 males and in females of strains BALB/cByJ, B6 and DBA2/J. Additionally, in that study heart rate was significantly reduced in B6 mice at the highest density [13]. An especially intriguing finding in the multi-strain study was that percent body fat increased with increasing density in strain 129, although it did not differ in B6.

In the current study we tested the effects of housing density in commonly used C57BL/6J x 129S1/SvImJ F1 hybrid mice (B6129SF1). This hybrid strain was selected because both parental strains had been tested for the effects of density previously [13]. We tested cage densities that were 0.5, 0.8, 1.2, and 1.8 times as dense as recommended in the *Guide* for mice weighing up to 25 g. In contrast to previous density studies in which mice were observed for up to 8 months of age, this study followed mice until they were 20 months of age. We measured survival, growth, behavior, body composition, blood pressure, clinical blood chemistries, immune function, mortality, and organ weights (testes, kidney, heart and adrenal glands). We did not study traits that did not differ among density groups in our previous studies. Immune function, known to be affected by chronic stress, has not previously been evaluated in our density studies. Our data revealed that B6129SF1 hybrid mice showed few statistically significant differences associated with density; males housed at higher density had reduced lean tissue mass and greater body fat and slightly elevated heart rate. Of 24 parameters measured in this study, we found no other significant differences that were associated with density.

## Materials and Methods

Hybrid female and male mice from a cross between C57BL/6J (B6) x 129S1/SvImJ (129) mice (B6129SF1/J; JAX stock no. 101043) were obtained at wean age from The Jackson Laboratory. Mice were provided *ad libitum* access to autoclaved acidified water (pH 2.8 to 3.1) and fed autoclaved standard laboratory chow containing 6% fat by weight (LabDiet^®^ 5K52, St. Louis, MO). The animal room was maintained at 22 ± 2°C, a humidity of 35% ± 4%, and a 12:12 hour light:dark cycle beginning at 0600. The animal room was supplied with HEPA-filtered air at 19 air changes per hour; each animal cage was individually ventilated at 60 air changes per hour. Bedding, changed weekly, was autoclaved pine shavings (Crobb Box, Ellsworth, ME). Mice were housed in “shoebox” cages (Thoren #5; “expanded mouse cage”) with 78.1 in^2^ (503.7 cm^2^) floor area. The dimensions for the #5 cage are 8.75 x 12.12 x 6.40 in (22.2 x 30.8 x 16.2 cm). The specific pathogen free animal room at the Jackson Laboratory was free of 15 viruses, 17 bacterial species, 2 *Mycoplasma* spp., external and internal parasites, and *Encephalitozoon cuniculi* [12]. At the end of the study, when mice were 20 months of age, mice were euthanized by CO_2_ exposure, unless tissues were to be harvested for weight or flow cytometry, in which case animals were euthanized by cervical dislocation. During the course of the study, 23 animals died by natural death and were discovered during routine daily welfare monitoring. No signs of suffering or ill health were observed in these animals prior to our having discovered that they had died. We use a body condition scoring system to evaluate health of all mice [20]. No animals in this study were observed with body condition scores less than BC3 (well-conditioned). All procedures were approved by the Institutional Animal Care and Use Committee at The Jackson Laboratory and are consistent with the United States Public Health Policy on the Humane Care and Use of Laboratory Animals.

Upon receipt at wean age, mice were randomly assigned to one of 4 density groups: 3, 5, 8 or 12 mice per cage. These density groups provide floor space per mouse that is 26.0, 15.6, 9.8, and 6.5 in^2^, corresponding to 0.5, 0.8, 1.2, and 1.8 times as dense as that recommended by the *Guide*, respectively. Each density group had 6 replicate cages per sex for a total of 48 cages and 336 mice in the experiment. In addition to routine welfare monitoring, mice were observed 3 times a week for mortality, morbidity, aggressive behavior (fighting, tail biting), compulsive behavior (whisker-picking, barbering) and stress (alopecia, body condition). Our study lasted until mice were 20 months of age. Near the end of the study, mice were randomly chosen from each density group to obtain physiological measurements. We drew from our experience in establishing a broad-based clinical mouse phenotyping facility [21, 22] to select the measurements used in this study and in our previous housing density studies. We included parameters known to vary in response to chronic or acute stressors, such as blood pressure, plasma glucose, immune function and adrenal weight, as well as other readily acquired parameters that may reveal additional variation in response to stress, such as body composition and additional plasma chemistries and organ weights. For phenotypic assessments, all 3 mice were sampled from the Density 3 group, and 5 mice were sampled from each of the Density 5, 8, and 12 groups. Some parameters used all 6 replicate cage sets, and some parameters (body composition, clinical chemistries, and blood pressure) used only 5 sets. Therefore, the number of mice used for each analysis differs slightly. Necropsy for organ weights and spleen harvest was performed on 3 mice per group.

Phenotyping for assessment of physiological parameters began one month prior to the end of the study, whereby a single test was performed in each of 4 successive weeks. First, blood pressure was measured using the tail-cuff method with unanesthetized mice as previously described [21] (Visitech Systems, Apex, NC). Briefly, mice were trained to the equipment for 2 days prior to acquiring measurements for analysis in the next 2 days. Measurements obtained were systolic and diastolic blood pressure and heart rate. Body composition (lean and fat tissue content, bone mineral density) was measured in the following week using dual-energy x-ray absorptiometry (DEXA; PIXImus II, GE- Lunar, Madison WI) under tribromoethanol anesthesia. One week later, blood was drawn via the retro-orbital sinus after a 4-hour food removal period for clinical blood chemistry assessments (glucose, triglycerides, urea, total protein, bilirubin, calcium, electrolytes). Clinical plasma chemistries were measured from EDTA-anticoagulated blood using a Beckman AU680 analyzer according to manufacturer’s specifications (Beckman Coulter, Fullerton, CA). Necropsy was performed the following week to weigh adrenal glands, kidneys, heart, and testes, and to harvest spleens for cytometric analysis and evaluation of immune function. Body weight for all mice was recorded monthly, using an Ohaus scale with InCal calibration to account for animal movement.

Statistical analysis was carried out separately for females and males because sex affects some traits. Randomly selected mice from each cage were measured as the technical replicates, using 3 mice from the Density 3 groups and 5 mice from all other density groups, and the measurements were averaged to obtain a cage mean value. Mortality, growth, behavior, immune function, and organ weight data were collected from each of 6 replicate cages per density. To accommodate workflow, only 5 replicate cages were sampled per density group for body composition, blood pressure and plasma chemistries. For each sex and density group, the replicate cage means, providing the biological replicates, were averaged and reported as mean ± standard error (SEM). A Fisher’s exact test was used to assess whether mortality was dependent on density. The software for statistical modeling was SAS v9.3 and JMP v10.0.0, both from SAS Institute (Cary, NC), and R v2.15.0 [23].

## Results / Discussion

Our study evaluated the effects of housing density on mortality, growth, body composition, behavior, blood pressure, clinical chemistries, immune function, and organ weights in B6129SF1 hybrid females and males. Of 24 parameters measured, we found that only 3 showed a significant difference related to density.

### Mortality

**Fig 1:**
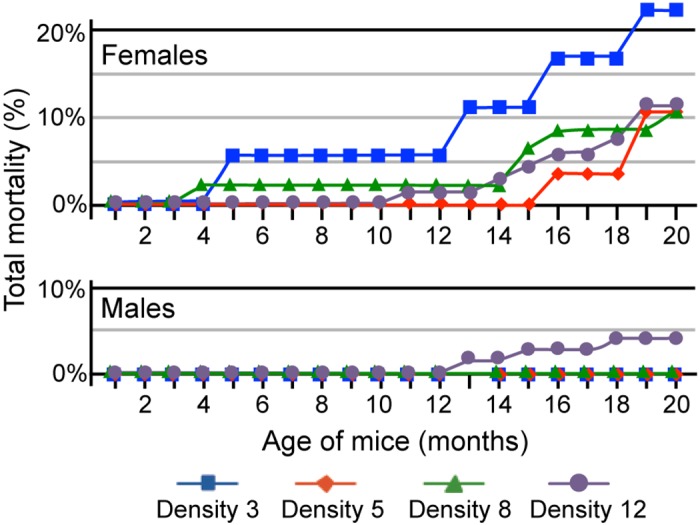
Total mortality by sex and density group throughout the study. Mortality of females (upper panel) was 12% overall, with the greatest mortality occurring in Density group 3 (22%; 4 of 18 animals). Mortality of males (lower panel) was 2% overall (3 of 168 animals); all deaths occurred in Density group 12.

Mortality of mice was followed in each group throughout the study (Fig 1). Mortality of females was 12% overall (20 of 168 mice). The highest mortality for females was in Density group 3 (4 of 18; 22%). In this group, the first animal died at 5 months of age; the next death did not occur until mice were 12 months of age. Mortality in the other female density groups was as follows: Density group 5, 3 of 30 (10%); Density group 8, 5 of 48 (10%); Density group 12, 8 of 72 (11%). It is notable that, for females, the highest mortality occurred in the lowest density group, with 3 mice per cage. No significant differences in mortality were found among density groups for females. Mortality of males was 2% overall (3 of 168 mice), with the earliest death at 13 months of age. No deaths occurred in the cages containing 3, 5, or 8 males. Deaths did occur in cages containing 12 males (3 of 72; 4%), but this was not significantly different from the other density groups. Statistical analysis of mortality indicated that mortality was independent of density for females and males in this study. In our previous housing density studies, mice were housed for shorter durations; within those time frames, mortality rates in the current study are comparable. In our study of B6 mice housed for 9 months, no mortality was observed for any group [12]. In our study of 5 inbred strains, including B6 and 129, in which mice were housed for 3 or 8 months, no mortality was observed at 3 months for each of these strains in any densities. In the 8-month study, strain 129 had 1.3% mortality, distributed evenly among densities. B6 mice had 0.25% mortality after 8 months, due to a single death in the highest density group [13]. Therefore, increased mortality observed in the current study is due primarily to the increased duration of the study, in which mice were almost 2 years old by the end.

### Growth

**Fig 2:**
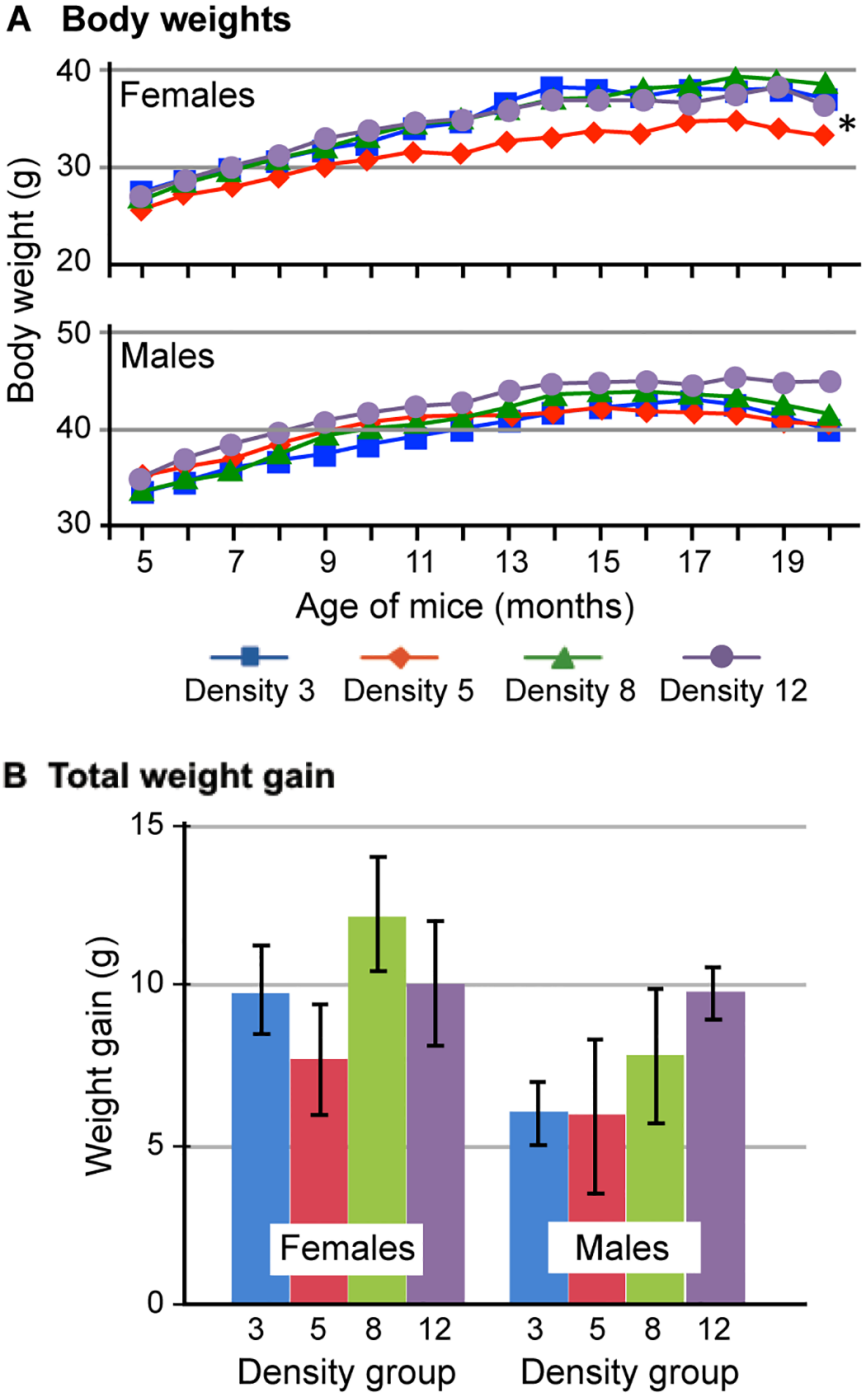
Growth by sex and density group from 5 to 20 months of age. (A) Growth curves from 5–20 months of age for female density groups (upper panel) and male density groups (lower panel). *For females, Density group 5 mice weighed significantly less than both the Density group 3 (P = 0.05) and Density group 8 (P = 0.04) animals. This difference was not observed until animals were 11 months old and was sustained for the duration of the study. No difference was found in growth among male density groups. (B) Total weight gained over the duration of the study did not differ significantly among density groups for females or males.

To assess growth, body weight was measured monthly from age 5 to 20 months. Reduced weight gain over time for a given density group may reflect an effect of chronic stress. Additionally, high variation in body weight within a group may indicate that a dominance hierarchy was established, suggesting that cage mates were chronically stressed and did not gain as much weight as a dominant mouse in the cage [24]. As expected, males weighed more than females throughout the study. For females, the only significant difference in growth among groups occurred in Density group 5 animals; this group weighed significantly less than both Density group 3 (P = 0.05) and Density group 8 (P = 0.04) animals (Fig 2A). Although this observation reached statistical significance, it does not indicate a trend that is consistent with lower or higher housing density. This difference was not observed until the 11-month time point and was sustained for the duration of the study. For males, body weight did not differ among density groups at any time point (Fig 2A). It is notable that standard deviations at each time point were low throughout the study. For example, body weight and SEMs for 12-month-old females in each density group were as follows: Density group 3 (34.9 ± 0.8 g); Density group 5 (31.4 ± 0.3 g); Density group 8 (34.9 ± 0.5 g); Density group 12 (35.3 ± 0.4 g). Body weight and SEMs for male density groups were as follows: Density group 3 (39.9 ± 1.3); Density group 5 (40.9 ± 1.8 g); Density group 8 (41.2 ± 0.9 g); Density group 12 (42.8 ± 0.5 g). Additionally, there was no significant difference among density groups for females or males in the total amount of weight gained over the duration of the study (Fig 2B).

### Behavior

Behavioral traits of barbering, whiskering and aggression (e.g., bite wounds) were recorded throughout the study (Table 1). Fighting was not observed in any density group. Almost all mice showed signs of barbering. Among females, barbering was common and equivalent in Density groups 5, 8, and 12, while less barbering occurred in Density group 3. Among males, barbering was common and equivalent in all density groups. Whiskering also occurred among all groups of mice in the study. Whiskering was frequent and occurred to the same extent in females and males of Density groups 5, 8, and 12. Slightly less whiskering occurred in Density group 3 for both sexes. Barbering and whiskering are usual behaviors observed in laboratory mice that vary in frequency among strains [25, 26]. This was also supported by observations in our previous study of 5 inbred strains, where these behaviors differed among strains but not among densities [13]. In that study, strain 129 was observed to barber significantly more than B6, but this observation was not density-dependent. Barbering behavior in the hybrid strain appears to reflect that of parental strain 129 rather than B6, based upon the high frequency of barbering observed in this study among all groups.

**Table 1:** Barbering and whiskering among density groups.

	Density group	Percentage of mice barbered (%)	Percentage of mice whiskered (%)
**Females**	**3**	**58**	**58**
	**5**	**94**	**94**
	**8**	**100**	**100**
	**12**	**100**	**100**
**Males**	**3**	**83**	**78**
	**5**	**100**	**94**
	**8**	**100**	**100**
	**12**	**94**	**100**

Percentages were derived from total instances over all cages per density group in which barbering occurred or in which whiskers had been chewed off.

Alopecia was not observed in this study of hybrid mice. In our previous study using parental strains 129 and B6, B6 showed more alopecia with increasing density at 8 months but not at 3 months, and strain 129 did not develop alopecia at any density or study duration [13]. Alopecia was minimal and not density-dependent in our previous study comparing densities of B6 housed together for 9 months [12].

Our results using barbering and whiskering to evaluate stress, taken together with results from our previous studies, suggest that these compulsive behaviors do not effectively describe endpoints of chronic stress but rather describe strain-specific behaviors. Likewise, we have previously demonstrated that alopecia also varies among inbred strains and is not correlated with cage density. In the current study, the absence of alopecia in the hybrid strain may simply reflect a dominant contribution of the trait from the 129 strain.

### Body composition

**Fig 3:**
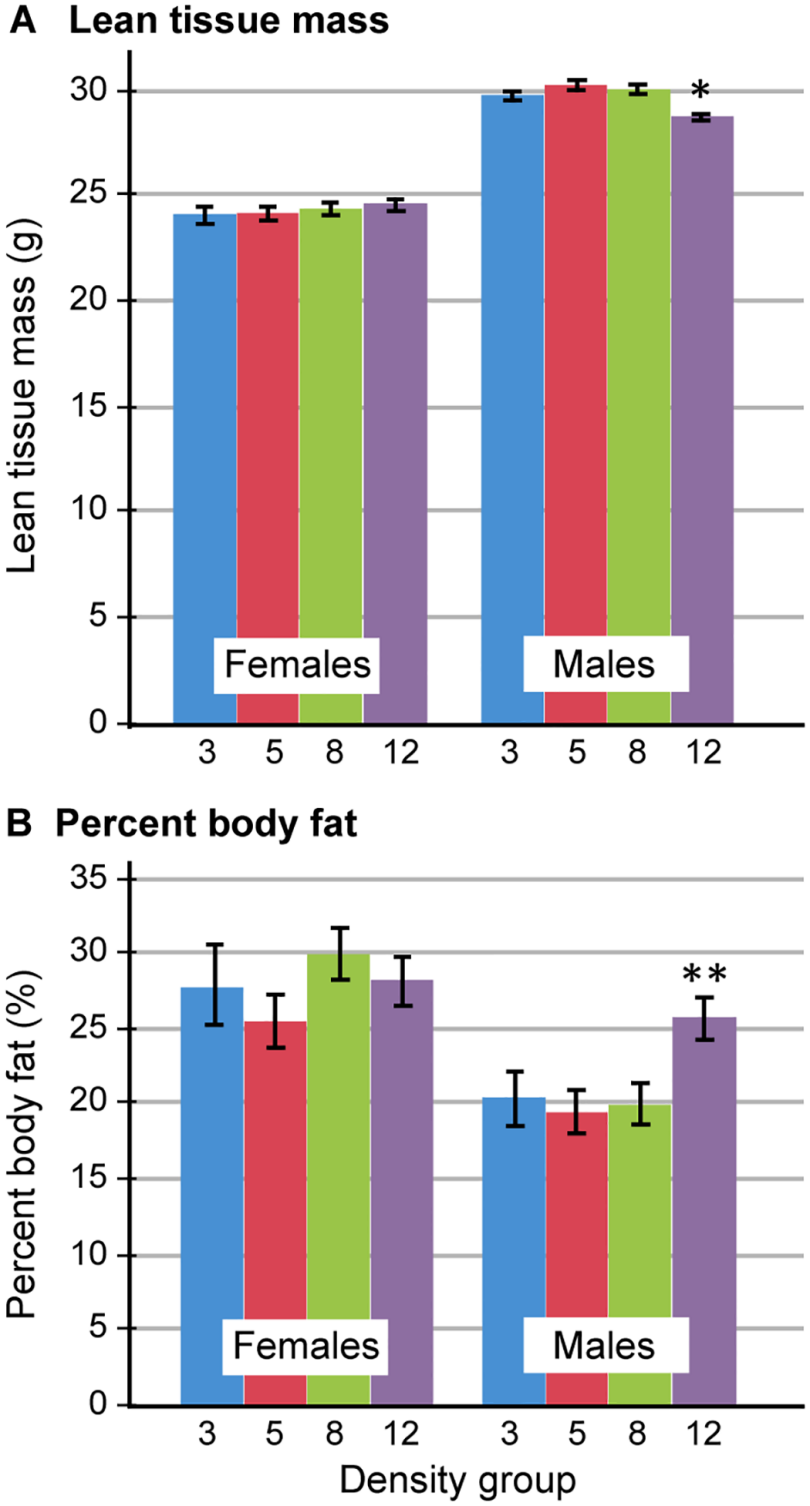
Body composition among females and males for all density groups. A) Lean tissue mass in grams, ± SEM. *Males of Density group 12 had significantly less lean mass than any other density group (P values of Density group 12 v. other groups are: v. Density group 3 = 0.02; v. Density group 5 = 0.0001; v. Density group 8 = 0.0008). B) Percent body fat ± SEM. **Males of Density group 12 had significantly more percent body fat than Density group 5 (P = 0.03) and Density group 8 (P = 0.01) but did not differ statistically from Density group 3.

Body composition, including bone mineral density, lean mass and percent body fat, was measured by DEXA 3 weeks before the end of the study. Bone mineral density did not differ among groups for females or males. Lean mass (LM) and percent body fat did not differ among densities for females (Fig 3). Density group 12 males had significantly less lean tissue (Fig 3A; mean LM = 28.8 ± 0.3g) than each of the other densities (Density group 3 males, P = 0.02, mean LM = 29.8 ± 0.3g; Density group 5 males, P < 0.0001, mean LM = 30.3 ± 0.3g; Density group 8 males, P = 0.0008, mean LM = 30.0 ± 0.3g). Likewise, Density group 12 males had significantly greater percent body fat (Fig 3B; mean body fat = 26.0 ± 1.4%) than Density group 5 males (P = 0.03, mean body fat = 19.8 ± 1.4%) and Density group 8 males (P = 0.01, mean body fat = 20.3 ± 1.4%) but did not differ statistically from Density group 3 males (mean body fat = 20.8 ± 1.8%). Higher percent body fat in Density group 12 males is consistent with the fact that these mice also had lower lean mass. The observation that males at the highest density had more body fat supports findings from our previous study of 5 inbred strains in which we found that males of strain 129, but not B6, had significantly more body fat than their counterparts at lower densities [13], and suggests that the hybrid strain mimics the 129 parent strain in this response. The higher body fat percent in the highest density group may reflect lower metabolic rate likely due to increased body temperature as a result of increased cage temperature in this group.

### Blood pressure

We measured systolic and diastolic blood pressure and heart rate in unanesthetized mice using the tail-cuff method, 2 weeks prior to the end of the study. For females, none of these parameters differed among density groups. For males, heart rate (HR) was slightly higher in Density group 12 mice (mean HR = 716 ± 7 bpm) than in Density group 3 (P = 0.01, mean HR = 679 ± 9 bpm) and Density group 5 mice (P = 0.0003, mean HR = 674 ± 7 bpm), but did not differ from Density group 8 mice (P = 0.06, mean HR = 691 ± 7 bpm). Systolic and diastolic blood pressure did not differ among male density groups. This result differs from our previous study of 5 inbred strains, in which HR was lower in the highest density group for B6 but did not differ for strain 129 [13]. The dissimilarity between the 2 studies could result from differences between them, namely the age at which mice were tested (8 months in the previous study versus 19 months in the present study), the number of mice per cage, the absolute mean values for HR, and the use of the hybrid strain in the current study.

### Clinical blood chemistries

While we have not seen significant differences in clinical blood chemistries measured in our previous housing density studies, we included certain assessments in the current study to determine whether they differed for the hybrid strain. We chose to measure parameters that have been shown to differ in other rodent studies evaluating effects of experimentally induced stress. Stressful experimental conditions such as severe food restriction or social isolation have been shown to impact clinical blood chemistries in rats [27, 28]. The only statistically significant difference among plasma clinical chemistries we found in this study was a slight decrease in bilirubin among Density group 8 males compared to Density group 3 (P = 0.02) and Density group 12 (P = 0.02). Mean bilirubin concentration for Density group 8 males did not differ from that of the Density group 5 males; hence, this difference did not follow a trend associated with density. Values for all clinical blood chemistries measured in our study can be found in S1 Table.

### Organ weights

Organ weights were measured in our previous studies. Among 5 inbred strains, as density increased, kidney weight was reduced in strain B6 but did not differ in strain 129, while adrenal weight decreased as density increased in 129 males [13]. To evaluate whether organ weights differed in B6129SF1 hybrid mice, weights of kidneys, heart, adrenal glands and testes were measured in the current study.

**Fig 4:**
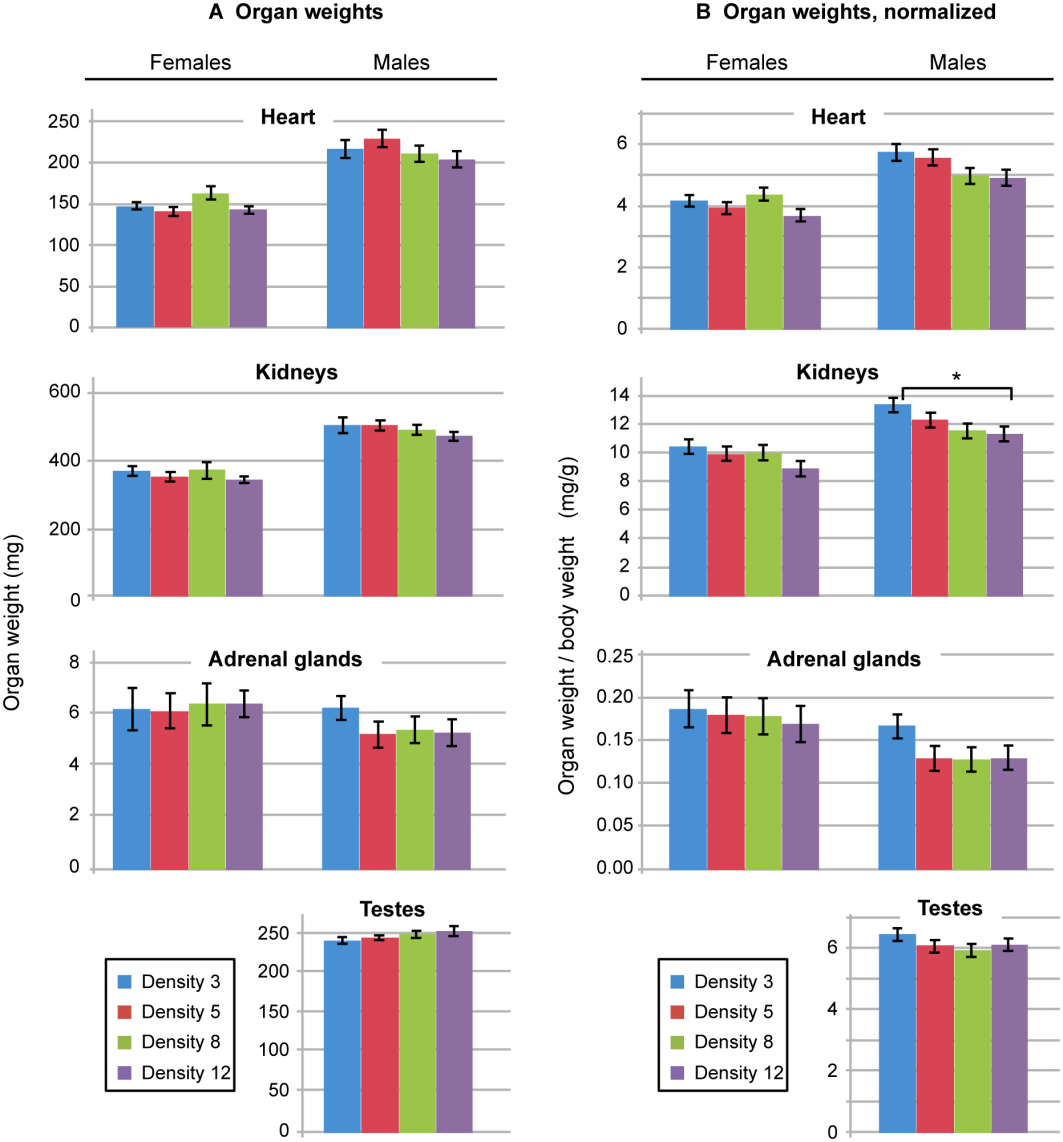
Organ weights by sex and density group. In panel A, absolute organ weights were analyzed; no differences for females or males were found. In panel B, organ weights were normalized to body weight. The only significant difference among density groups was for male kidney weight, in which Density group 3 male kidneys weighed more than Density group 12 male kidneys. Error bars are SEM. *P = 0.05; n = 3 animals per group.

We analyzed absolute organ weights as well as those normalized to body weight. No differences in absolute organ weights were found for females or males among density groups (Fig 4A). Because body weight for females of Density group 5 differed from other groups, we normalized the data to address this difference. Using values normalized to body weight, female values did not differ among density groups (Fig 4B); the only significant difference we found was mean kidney weight among Density group 3 males, which was slightly greater than that of Density group 12 males (P = 0.05). However, for males, analysis of absolute organ weight data may be more appropriate than analysis of normalized data. Male body weight did not differ among density groups (Fig 1A), but we did observe increased percent fat in Density group 12 males compared to Density group 5 and Density group 8 males (Fig 3B). This change in body composition, but not in weight, warrants evaluation of non-normalized absolute organ weights so that adiposity does not confound the analysis.

### Immune function

**Fig 5:**
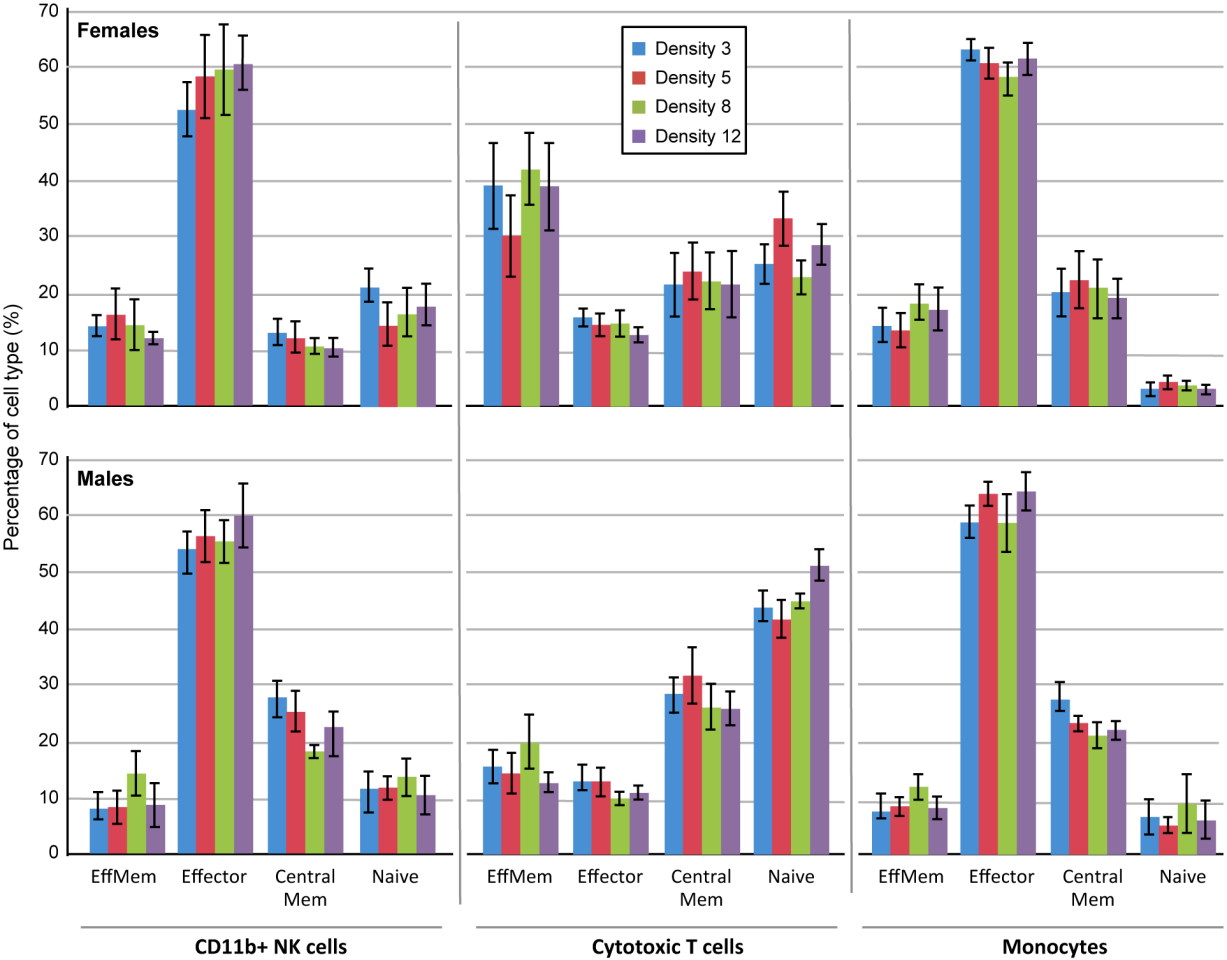
Flow cytometric analysis of spleens. No significant difference was found among density groups for females or males for any of the 12 immune cell populations and their subpopulations measured in the current study. CD11b+ NK cells, cytotoxic T cells, and monocytes were chosen to represent cytometric data obtained in our study based upon previous studies that found significant differences in these populations in response to experimental stressors (see text). Top panel shows female data; bottom panel shows male data. Subpopulation abbreviations: EffMem = effector memory cells; CentralMem = central memory cells.

Numerous studies evaluating the impact of chronic and other psychosocial stressors on immune function have demonstrated adverse effects on both innate and adaptive immunity in the mouse [29–31]. We therefore included evaluation of immune cell populations by flow cytometry of spleens in the current study. We measured 12 immune cell populations including B cells, NK cells, T cells (helper, cytotoxic, gamma/delta), eosinophils, monocytes, and peripheral mononuclear cells. For each of these cell types, 4 subpopulations were measured: effector memory, effector, central memory, and naïve. The complete set of data for all immune cells measured is presented in S2 Table. We found no difference in any immune cell measurement among density groups. Fig 5 shows representative results of this analysis and includes immune cell populations shown by others to be affected by various experimental stressors: NK cells [29], CD8+ cytotoxic T cells [30], and CD11b+ monocytes [31]. We did not follow immune function longitudinally, so our results do not capture fluctuations that may have occurred prior to 20 months of age. Our results describe immune status at the end of the study only, and suggest that any fluctuations in immune cell profiles were resolved by the time mice were 20 months of age. Of 48 subpopulations measured, we found significant sex differences for 16 measurements (P ≤ 0.05; see S2 Table).

## Conclusion

In this study of hybrid mice, we found no significant differences in any measured parameters among female density groups, and density had an impact on only a few parameters among male groups. The differences in males were: reduced lean mass and increased percent body fat in the highest density group and slightly elevated heart rate in the 2 higher density groups. Increased body fat percent in males at the highest density may reflect a lower metabolic rate due to increased body temperature, resulting from increased cage temperature. This observation may also stem from a strain-specific difference in the 129 parental strain, as we previously observed increased percent body fat at higher density for strain 129 but not B6. Whether the subtle change in heart rate reflects a difference in well-being is unclear, because mean values in the hybrid strain are well within normal physiological ranges for each of the parental strains [32]. Overall, we have demonstrated that housing this hybrid strain at half to near twice the density currently recommended does not impair baseline physiology and well-being. Furthermore, a slight elevation in body fat seen in males at the highest density in this study supports our previous findings for the 129 strain and suggests that improved metabolic status may be a benefit of housing mice more densely.

These results add the B6129SF1 hybrid mouse strain to the increasing body of evidence demonstrating that commonly used inbred strains are not adversely affected by housing at increased density. However, outbred mice have not yet been thoroughly tested for density effects; such investigations would add valuable data to inform future optimal use of mice in biomedical research.

## Supporting Information

S1 Table Clinical blood chemistries evaluated across density groups. Values are least square mean ± standard error for each density group. *Significant differences were found in Density group 8 males compared to Density group 3 (P = 0.02) and Density group 12 (P = 0.02) males. BUN = blood urea nitrogen; Ca2+ = calcium, ionized; Na+ = sodium; Cl− = chloride; K+ = potassium; CO2 = carbon dioxide.
(DOCX)

S2 Table Immune cell populations evaluated across density groups. *Significant differences (P ≤ 0.05) were found in these sub-populations between sexes. EffMem = effector memory cells; CentralMem = central memory cells.
(DOCX)
