# Supplementary material for: Effects of Varied Housing Density on a Hybrid Mouse Strain Followed for 20 Months
Source: PLoS One. 2016 Feb 22;11(2):e0149647. doi: 10.1371/journal.pone.0149647 (PMC4762697; doi:10.1371/journal.pone.0149647)
Supplement: S1 Table — Values are least square mean ± standard error for each density group. *Significant differences were found in Density group 8 males compared to Density group 3 (P = 0.02) and Density group 12 (P = 0.02) males. BUN = blood urea nitrogen; Ca2+ = calcium, ionized; Na+ = sodium; Cl− = chloride; K+ = potassium; CO2 = carbon dioxide. (DOCX) [file pone.0149647.s001.docx]

**S1 Table. Clinical blood chemistries evaluated across density groups.**

| **Density group** | **Variable** | | | | | | | | | |
| --- | --- | --- | --- | --- | --- | --- | --- | --- | --- | --- |
|  | **Bilirubin* (mg/dL)** | **BUN (mg/dL)** | **Ca^2+^ (mg/dL)** | **Glucose (mg/dL)** | **Total protein (g/dL)** | **Triglycerides (mg/dL)** | **Na^+^ (mmol/L)** | **Cl^−^ (mmol/L)** | **K^+^ (mmol/L)** | **CO_2_ (mmol/L)** |
| Females |  |  |  |  |  |  |  |  |  |  |
| 3 | 0.21 ± 0.02 | 15.8 ± 1.0 | 9.1 ± 0.1 | 125 ± 5 | 5.8 ± 0.2 | 115.2 ± 11.7 | 146.9 ± 0.8 | 108.1 ± 0.8 | 6.1 ± 0.1 | 12.6 ± 0.4 |
| 5 | 0.21 ± 0.01 | 17.0 ± 0.8 | 9.2 ± 0.1 | 127 ± 4 | 5.6 ± 0.1 | 110.5 ± 8.8 | 146.5 ± 0.6 | 108.0 ± 0.6 | 6.3 ± 0.1 | 12.9 ± 0.3 |
| 8 | 0.22 ± 0.01 | 17.1 ± 0.8 | 9.2 ± 0.1 | 128 ± 4 | 5.8 ± 0.1 | 125.1 ± 9.4 | 145.6 ± 0.7 | 106.7 ± 0.6 | 6.1 ± 0.1 | 12.7 ± 0.3 |
| 12 | 0.19 ± 0.01 | 16.4 ± 0.7 | 9.3 ± 0.1 | 126 ± 4 | 5.6 ± 0.1 | 109.8 ± 8.8 | 147.4 ± 0.6 | 108.5 ± 0.6 | 5.9 ± 0.1 | 13.1 ± 0.3 |
| Males |  |  |  |  |  |  |  |  |  |  |
| 3 | 0.22 ± 0.01 | 16.1 ± 0.9 | 8.8 ± 0.1 | 125 ± 5 | 5.2 ± 0.2 | 163.5 ± 10.9 | 146.8 ± 0.8 | 107.0 ± 0.7 | 6.2 ± 0.1 | 12.8 ± 0.4 |
| 5 | 0.21 ± 0.01 | 18.4 ± 0.7 | 8.9 ± 0.1 | 128 ± 4 | 5.4 ± 0.1 | 165.1 ± 8.4 | 147.1 ± 0.6 | 106.7 ± 0.6 | 6.3 ± 0.1 | 12.6 ± 0.3 |
| 8 | 0.18 ± 0.01 | 18.6 ± 0.7 | 8.9 ± 0.1 | 120 ± 4 | 5.5 ± 0.1 | 156.1 ± 8.6 | 147.7 ± 0.6 | 107.1 ± 0.6 | 6.4 ± 0.1 | 13.1 ± 0.3 |
| 12 | 0.22 ± 0.01 | 19.2 ± 0.7 | 9.0 ± 0.1 | 129 ± 4 | 5.3 ± 0.1 | 149.8 ± 8.4 | 147.1 ± 0.6 | 106.2 ± 0.5 | 6.4 ± 0.1 | 12.5 ± 0.3 |

Values are least square mean ± standard error for each density group. *Significant differences were found in Density group 8 males compared to Density group 3 (P = 0.02) and Density group 12 (P = 0.02) males. BUN = blood urea nitrogen; Ca^2+^ = calcium, ionized; Na^+^ = sodium; Cl**^−^** = chloride; K^+^ = potassium; CO_2_ = carbon dioxide.
