# Supplementary material for: Effects of Varied Housing Density on a Hybrid Mouse Strain Followed for 20 Months
Source: PLoS One. 2016 Feb 22;11(2):e0149647. doi: 10.1371/journal.pone.0149647 (PMC4762697; doi:10.1371/journal.pone.0149647)
Supplement: S2 Table — *Significant differences (P ≤ 0.05) were found in these sub-populations between sexes. EffMem = effector memory cells; CentralMem = central memory cells. (DOCX) [file pone.0149647.s002.docx]

**S2 Table. Immune cell populations evaluated across density groups.**

| **Variable** | | **Mean value (± SEM)** | | | | | | | | |
| --- | --- | --- | --- | --- | --- | --- | --- | --- | --- | --- |
|  |  | **Females** | | | |  | **Males** | | | |
|  |  | **Density group 3** | **Density group 5** | **Density group 8** | **Density group 12** |  | **Density group 3** | **Density group 5** | **Density group 8** | **Density group 12** |
| Bcells | EffMem | 23.1 ± 7.8 | 27.6 ± 8.7 | 27.0 ± 9.0 | 28.3 ± 8.8 |  | 27.5 ± 12.3 | 31.0 ± 13.7 | 35.7 ± 13.2 | 31.1 ± 13.5 |
|  | Effector | 8.0 ± 2.6 | 6.3 ± 0.6 | 10.9 ± 3.2 | 9.3 ± 2.6 |  | 7.9 ± 3.1 | 6.6 ± 1.3 | 6.7 ± 2.2 | 6.7 ± 0.9 |
|  | CentralMem* | 10.5 ± 2.5 | 9.8 ± 2.0 | 9.6 ± 2.1 | 7.2 ± 0.9 |  | 13.1 ± 1.4 | 13.3 ± 1.6 | 9.5 ± 1.6 | 15.1 ± 1.6 |
|  | Naive | 58.5 ± 10.4 | 56.2 ± 10.2 | 52.5 ± 10.5 | 55.2 ± 10.7 |  | 51.5 ± 14.8 | 49.1 ± 14.1 | 48.2 ± 14.6 | 47.0 ± 13.7 |
| CD11b+B-1_Bcells | EffMem* | 14.3 ± 5.9 | 12.0 ± 2.8 | 15.9 ± 3.6 | 15.8 ± 3.7 |  | 4.0 ± 0.9 | 5.0 ± 1.5 | 11.0 ± 4.2 | 4.9 ± 1.3 |
|  | Effector | 27.9 ± 1.4 | 24.9 ± 2.7 | 29.0 ± 3.0 | 26.5 ± 1.2 |  | 21.6 ± 5.2 | 25.2 ± 6.0 | 21.8 ± 3.4 | 24.4 ± 5.7 |
|  | CentralMem* | 40.1 ± 7.5 | 45.4 ± 5.5 | 37.5 ± 6.8 | 38.9 ± 5.1 |  | 56.8 ± 2.2 | 55.0 ± 4.6 | 47.7 ± 5.7 | 55.3 ± 2.3 |
|  | Naive | 17.7 ± 2.0 | 17.7 ± 1.9 | 17.6 ± 2.5 | 18.8 ± 1.4 |  | 17.5 ± 4.7 | 14.8 ± 4.4 | 19.6 ± 4.6 | 15.4 ± 4.3 |
| CD11b+_NK | EffMem* | 14.0 ± 1.9 | 16.0 ± 4.4 | 14.2 ± 4.4 | 11.9 ± 1.1 |  | 7.6 ± 1.9 | 7.9 ± 2.9 | 13.9 ± 3.9 | 8.3 ± 3.9 |
|  | Effector | 52.0 ± 4.9 | 57.7 ± 7.4 | 58.9 ± 8.0 | 60.1 ± 4.8 |  | 53.5 ± 4.3 | 55.7 ± 4.5 | 54.7 ± 3.6 | 59.3 ± 5.6 |
|  | CentralMem | 12.9 ± 2.2 | 11.9 ± 2.6 | 10.5 ± 1.4 | 10.2 ± 1.7 |  | 27.3 ± 3.5 | 24.9 ± 3.6 | 17.8 ± 1.2 | 22.1 ± 2.7 |
|  | Naive* | 21.1 ± 2.9 | 14.3 ± 3.8 | 16.4 ± 4.2 | 17.7 ± 3.6 |  | 11.5 ± 4.3 | 11.5 ± 2.0 | 13.5 ± 3.4 | 10.2 ± 3.3 |
| CD11b-NK | EffMem* | 20.5 ± 4.6 | 23.1 ± 4.8 | 21.4 ± 3.9 | 20.6 ± 2.9 |  | 12.3 ± 2.1 | 10.1 ± 1.9 | 20.7 ± 5.3 | 9.1 ± 1.7 |
|  | Effector* | 48.4 ± 4.8 | 50.0 ± 1.8 | 53.4 ± 2.6 | 51.5 ± 3.1 |  | 43.8 ± 0.9 | 47.6 ± 2.3 | 41.7 ± 1.9 | 46.0 ± 3.0 |
|  | CentralMem | 20.7 ± 3.9 | 20.7 ± 4.5 | 17.2 ± 3.6 | 17.7 ± 2.2 |  | 38.0 ± 2.9 | 36.7 ± 4.6 | 29.9 ± 4.4 | 37.9 ± 2.0 |
|  | Naive | 10.4 ± 2.6 | 6.2 ± 0.7 | 8.0 ± 1.8 | 10.2 ± 0.9 |  | 6.0 ± 0.8 | 5.6 ± 1.1 | 7.7 ± 1.2 | 7.1 ± 2.2 |
| NK-T | EffMem* | 21.6 ± 4.5 | 24.1 ± 5.0 | 23.1 ± 3.2 | 25.0 ± 2.8 |  | 12.7 ± 2.6 | 10.1 ± 1.9 | 19.9 ± 5.7 | 10.6 ± 1.3 |
|  | Effector* | 63.8 ± 2.4 | 61.6 ± 3.3 | 64.9 ± 2.9 | 63.2 ± 2.4 |  | 56.4 ± 2.2 | 60.5 ± 2.6 | 52.8 ± 3.5 | 57.7 ± 2.8 |
|  | CentralMem | 10.4 ± 2.7 | 9.9 ± 2.6 | 8.3 ± 2.2 | 8.1 ± 1.6 |  | 20.2 ± 1.3 | 20.4 ± 2.6 | 18.4 ± 2.7 | 18.3 ± 0.7 |
|  | Naive | 4.2 ± 0.8 | 4.4 ± 0.9 | 3.7 ± 0.8 | 3.6 ± 0.7 |  | 10.7 ± 1.3 | 9.0 ± 0.7 | 8.9 ± 1.0 | 13.4 ± 1.6 |
| HelperT | EffMem | 45.7 ± 6.7 | 41.3 ± 5.8 | 47.0 ± 5.8 | 47.7 ± 2.8 |  | 26.8 ± 4.4 | 25.8 ± 4.4 | 36.3 ± 6.0 | 24.4 ± 2.5 |
|  | Effector | 46.2 ± 5.7 | 48.9 ± 4.1 | 46.1 ± 4.9 | 45.6 ± 2.6 |  | 48.0 ± 3.2 | 51.0 ± 4.2 | 43.5 ± 3.2 | 48.5 ± 3.7 |
|  | CentralMem | 2.8 ± 0.9 | 3.6 ± 0.8 | 2.5 ± 0.6 | 1.9 ± 0.2 |  | 6.5 ± 0.6 | 6.4 ± 1.1 | 4.5 ± 0.9 | 6.4 ± 0.4 |
|  | Naive | 5.2 ± 1.0 | 6.2 ± 1.1 | 4.4 ± 0.9 | 4.7 ± 0.3 |  | 18.7 ± 1.4 | 16.9 ± 2.0 | 15.7 ± 2.1 | 20.8 ± 1.8 |
| Cytotoxic_T | EffMem | 38.4 ± 7.6 | 29.5 ± 7.1 | 41.3 ± 6.3 | 38.2 ± 7.7 |  | 15.5 ± 3.0 | 14.3 ± 3.6 | 19.8 ± 4.7 | 12.7 ± 1.7 |
|  | Effector | 15.4 ± 1.6 | 14.1 ± 2.1 | 14.3 ± 2.3 | 12.3 ± 1.3 |  | 13.0 ± 1.7 | 12.9 ± 2.5 | 9.9 ± 1.2 | 11.0 ± 1.3 |
|  | CentralMem | 21.2 ± 5.7 | 23.4 ± 4.9 | 21.7 ± 5.0 | 21.2 ± 5.9 |  | 28.2 ± 3.3 | 31.5 ± 4.9 | 25.8 ± 4.2 | 25.6 ± 2.9 |
|  | Naive | 25.1 ± 3.5 | 33.1 ± 4.8 | 22.7 ± 2.9 | 28.4 ± 3.6 |  | 43.4 ± 2.4 | 41.3 ± 3.4 | 44.4 ± 1.3 | 50.7 ± 2.8 |
| gd_T | EffMem* | 41.3 ± 7.0 | 35.7 ± 6.8 | 49.1 ± 6.9 | 48.6 ± 6.1 |  | 24.2 ± 5.0 | 23.5 ± 5.2 | 33.7 ± 8.3 | 23.2 ± 3.5 |
|  | Effector | 33.5 ± 4.4 | 33.1 ± 5.6 | 27.9 ± 5.5 | 27.9 ± 5.2 |  | 36.1 ± 3.7 | 38.9 ± 4.9 | 29.9 ± 5.3 | 40.5 ± 3.2 |
|  | CentralMem | 8.3 ± 3.3 | 9.2 ± 3.0 | 6.6 ± 2.8 | 4.6 ± 2.0 |  | 18.8 ± 2.9 | 16.8 ± 3.6 | 16.2 ± 5.0 | 15.7 ± 2.7 |
|  | Naive | 16.9 ± 2.7 | 22.0 ± 1.9 | 16.4 ± 2.3 | 19.0 ± 1.6 |  | 20.9 ± 1.5 | 20.6 ± 2.9 | 20.2 ± 2.6 | 20.6 ± 1.4 |
| Eosinophils | EffMem* | 12.0 ± 5.8 | 6.8 ± 2.7 | 8.8 ± 3.1 | 10.9 ± 4.5 |  | 3.3 ± 1.1 | 3.3 ± 1.5 | 3.8 ± 1.8 | 6.1 ± 2.2 |
|  | Effector | 53.2 ± 4.7 | 61.2 ± 4.3 | 53.2 ± 3.0 | 51.7 ± 3.1 |  | 54.0 ± 4.1 | 51.6 ± 4.5 | 54.8 ± 4.7 | 45.2 ± 3.5 |
|  | CentralMem* | 34.1 ± 7.5 | 31.4 ± 6.4 | 37.8 ± 5.5 | 36.7 ± 6.2 |  | 42.4 ± 4.3 | 45.2 ± 5.1 | 40.8 ± 6.7 | 48.2 ± 3.8 |
|  | Naive | 0.7 ± 0.4 | 0.4 ± 0.2 | 0.5 ± 0.2 | 0.7 ± 0.2 |  | 0.4 ± 0.3 | 0.3 ± 0.2 | 0.6 ± 0.4 | 0.5 ± 0.4 |
| Monocytes | EffMem* | 14.2 ± 3.1 | 13.3 ± 3.2 | 18.1 ± 3.2 | 17.0 ± 3.8 |  | 7.5 ± 1.3 | 8.3 ± 1.7 | 11.7 ± 2.3 | 8.1 ± 1.9 |
|  | Effector | 62.6 ± 1.9 | 60.1 ± 2.9 | 57.4 ± 3.0 | 60.9 ± 2.8 |  | 58.6 ± 2.8 | 63.6 ± 2.1 | 58.4 ± 5.0 | 64.1 ± 3.3 |
|  | CentralMem | 20.0 ± 4.1 | 22.2 ± 5.1 | 20.7 ± 5.2 | 19.0 ± 3.6 |  | 27.2 ± 2.1 | 23.1 ± 1.4 | 20.9 ± 2.3 | 21.8 ± 1.7 |
|  | Naive | 3.2 ± 1.3 | 4.3 ± 1.1 | 3.9 ± 0.9 | 3.1 ± 1.0 |  | 6.7 ± 3.2 | 5.1 ± 1.4 | 8.9 ± 5.2 | 6.0 ± 3.5 |
| PMN | EffMem* | 1.4 ± 0.7 | 1.0 ± 0.4 | 1.6 ± 0.6 | 0.8 ± 0.3 |  | 0.3 ± 0.2 | 0.1 ± 0.1 | 0.6 ± 0.2 | 0.1 ± 0.1 |
|  | Effector | 31.4 ± 4.0 | 29.8 ± 3.4 | 38.3 ± 5.2 | 31.6 ± 4.0 |  | 30.9 ± 5.8 | 28.4 ± 3.1 | 32.3 ± 7.0 | 26.0 ± 3.0 |
|  | CentralMem | 66.9 ± 4.5 | 68.9 ± 3.6 | 59.6 ± 5.6 | 66.8 ± 4.1 |  | 68.8 ± 5.9 | 71.5 ± 3.2 | 67.0 ± 7.2 | 73.9 ± 3.1 |
|  | Naive* | 0.4 ± 0.2 | 0.4 ± 0.2 | 0.5 ± 0.2 | 0.7 ± 0.4 |  | 0.0 ± 0.0 | 0.0 ± 0.0 | 0.1 ± 0.0 | 0.0 ± 0.0 |
| Unidentified | EffMem | 49.2 ± 4.7 | 56.2 ± 2.1 | 60.3 ± 3.2 | 61.1 ± 3.4 |  | 60.2 ± 2.5 | 58.6 ± 3.0 | 63.5 ± 2.8 | 60.3 ± 1.9 |
|  | Effector | 42.7 ± 6.0 | 35.0 ± 1.5 | 30.8 ± 2.4 | 32.0 ± 2.9 |  | 34.2 ± 2.7 | 36.0 ± 2.7 | 28.9 ± 2.0 | 32.8 ± 1.5 |
|  | CentralMem | 6.0 ± 1.8 | 6.2 ± 2.1 | 6.4 ± 1.5 | 4.4 ± 0.8 |  | 4.7 ± 0.8 | 4.4 ± 0.4 | 4.9 ± 1.7 | 6.3 ± 1.6 |
|  | Naive* | 2.1 ± 0.9 | 2.6 ± 0.6 | 2.6 ± 0.8 | 2.5 ± 0.5 |  | 0.9 ± 0.2 | 1.0 ± 0.3 | 2.6 ± 1.5 | 0.7 ± 0.1 |

*Significant differences (P ≤ 0.05) were found in these sub-populations between sexes. EffMem = effector memory cells; CentralMem = central memory cells.
